# Investigations for Design Estimation of an Anisotropic Polymer Matrix Composite Plate with a Central Circular Hole under Uniaxial Tension

**DOI:** 10.3390/polym14101977

**Published:** 2022-05-12

**Authors:** Seongsik Lim, Vivek Kumar Dhimole, Yongbae Kim, Chongdu Cho

**Affiliations:** 1 Metal Forming R&D Department, Korea Institute of Industrial Technology (KITECH), Incheon 21999, Korea; sslim@kitech.re.kr (S.L.); yb_kim@kitech.re.kr (Y.K.); 2 Mechanical Engineering Department, Inha University Graduate School, Incheon 22212, Korea; vivek.dhimole@inha.edu

**Keywords:** plate with hole, carbon/epoxy polymer, braided composites, finite element analysis, stress behavior, stress concentration factor (SCF), shear deformation theories, thickness aspect

## Abstract

Composite plates with holes are common in engineering applications, such as the automotive and aerospace industries. Three-dimensional braided carbon/epoxy polymers are an advanced textile composite and are used in various structures due to their high damage resistance and relatively low manufacturing cost. When a braided polymer plate with a hole is used in engineering applications, it is necessary to know its mechanical behavior under loading conditions using analysis theory to design it better. However, the effects of stress distribution with shear deformation theories on the variable thickness of the braided polymer plate (carbon/epoxy) with a hole under tensile loading have not been reported yet. In this paper, a study is conducted to evaluate shear deformation theories for a braided polymer plate with variable thickness and a hole in the center, analyzing the stresses and their concentration variations. First, multiscale modeling and analysis are performed to determine the mechanical properties of the plate. Then, finite element analyses are performed on a homogenized macro plate with a hole. The analysis process is verified by comparison with the available literature. Results show that the first-order shear deformation theory calculates 37, 56, and 70 percent less maximum transverse shear stress than the high-order shear deformation theory (Reissner–Mindlin) and the elasticity theory for thin, moderately thick, and thick braided polymer plates, respectively. Additionally, changing the theory has no significant effect on circumferential stress, radial stress, Von Mises stress, and stress concentration factor. As a result, this research can provide researchers and designers with structural intuition for a braided polymer plate with a center hole.

## 1. Introduction

The 3D braided polymer composites are advanced textile polymer composites. They have a wide range of excellent mechanical properties, such as high out-of-plane strength, excellent damage and impact resistance due to the outstanding internal structure of 3D braided preforms. This leads to the widespread use of 3D braided composites in different fields, such as aeronautics, marine, transportation, and other industries. Different holes or openings are typically drilled into these composites’ plates to reduce the system’s weight and allow access to system equipment [1]. However, due to specified cut-outs, significant stresses are formed around the holes or openings when a plate is subjected to tension or shear pressure. Therefore, an accurate knowledge of stresses and stress concentration is essential for designing such plates with holes. Due to time and cost issues in the experimental methods, researchers are focusing on numerical and analytical methods [2].

Researchers have analyzed different conditions for a plate with a hole. Studies related to isotropic plates with a hole have been covered widely in the past literature [3]. Afterward, considerations have also shifted toward orthotropic and laminated plates with holes. Hwai Chung and Bin [4] established an empirical model to compute the stress concentration factor (SCF) for isotropic/orthotropic plates with circular holes. Due to the difficulty in obtaining the negative SCFs analytically, the suggested technique could not consistently forecast the SCFs of orthotropic plates and cylinders under biaxial loads. Under the varied transverse static loading conditions, Jain and Mittal [5] studied the influence of hole diameter to plate width on SCF and deflection in isotropic, orthotropic, and laminated composite plates. Mittal and Jain [6] also used two-dimensional finite element methods to investigate the effect of fiber orientation on the SCF in a fibrous plate with a central circular hole under transverse static loading. Their work is limited to case studies, such as symmetric and antisymmetric laminates, and then fiber orientation effect on laminated and woven composites. Toubal et al. [7] investigated the stress concentration in a circular composite plate with a hole, and the aim was to focus on only the circular plate and its stress concentration. Hashem et al. [8] calculated the SCF for arbitrarily oriented irregular fiber laminas with square/circular holes using a numerical model. The work was based on Howland and Heywood and mainly focused on a plate with a center square hole. Lekhnitskii et al. [9] and Tan [10] proposed various formulations to investigate stress concentration for infinite and finite orthotropic plates. Nicholas and Christoph [11] investigated the stress concentration variables for cylindrically orthotropic plates. The study focused on a circular orthotropic plate with a central circular hole. Additionally, an experimental approach based on digital image correlation was reported by Mhallah and Bouraoui [12] to determine the SCF for orthotropic and isotropic materials. Wang et al. [13] investigated a functionally graded plate with an elliptical hole under tensile load and found the inhomogeneity effect on the stress concentration and damage factor compared to homogeneous materials. Apart from that, researchers have also analyzed the plate with a center hole under heat load. Chaleshtari et al. [14] analyzed a symmetric laminate plate with a rectangular hole under heat flux loads and investigated the effect of hole rotation and heat flux angle on thermal stress around the hole. Recently, Zappino et al. [15] proposed an experimental and numerical study to see the capabilities of additive manufactured composite materials to reduce stress and strain concentrations in open-hole plates. The study was conducted on isotropic and anisotropic plates with a hole. So far, most of the studies considered plates with a different kind of hole for stress distribution around the hole and calculated the effect of plate length versus the diameter of the hole with isotropic and orthotropic laminates. The literature shows that a design study of braided polymer plate with a hole in terms of stress and its concentration factor has not yet been investigated. Further, the shear deformation theory effect on variable thicknesses has also not been reported in the existing literature.

Therefore, the current research work presents stress behavior with the effectiveness of analysis theories for variable thicknesses of a 3D braided polymer composite plate. Firstly, multiscale modeling of the braided composite plate is conducted. Then, the analysis process is verified and extended to the currently considered plate. Under various conditions, the results are presented for circumferential, radial, and transverse shear stresses. Additionally, SCF is determined for polymer braided composite plate concerning plate width/hole diameter (w/d). As analytic treatment of such a problem is highly challenging, the finite element approach is used for the entire study.

## 2. Modeling and Analysis

### 2.1. Modeling

**Figure 1:**
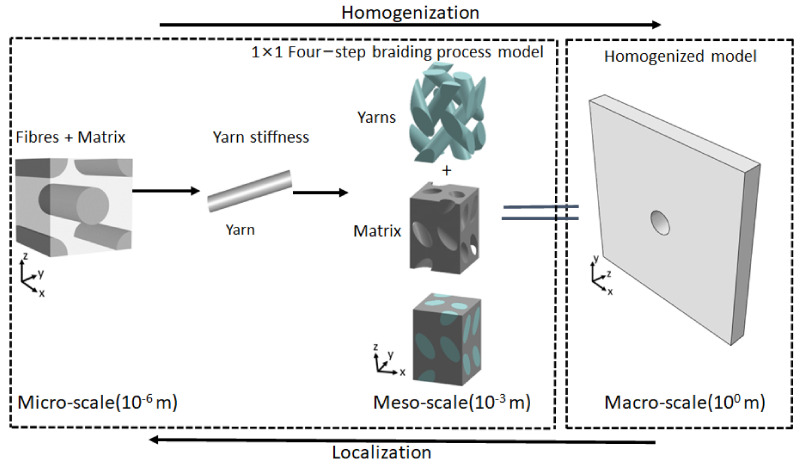
Multiscale modeling for braided polymer plate.

The braided anisotropic plate with a center hole is modeled for investigation. Due to heterogeneity, three-scale modeling is performed, namely micro, meso, and macro, as shown in Figure 1. At the micro and meso levels, homogenization is performed to calculate mechanical stiffness for the macro plate in order to analyze it in Section 2.2. 

**Figure 2:**
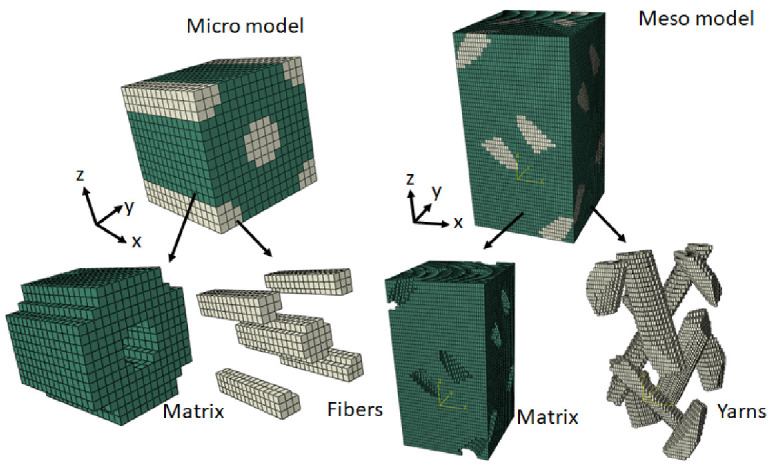
Micro- and mesoscale models.

First, the yarn properties are calculated in microscale homogenization based on fiber distribution. The main constituents are epoxy resin and T300 carbon fiber, as shown in Table 1. The hexagonal distribution is adopted on a microscale to cover accurate details. Then, the unit cell model based on the yarn trajectory is built in the mesoscale based on a 1*1 four-step braiding manufacturing process [16]. This 1*1 four-step braiding process forms the braided composite by a rectangular preform in a cyclic process, so yarns are distributed uniformly across the plate and around the hole. Therefore, uniformity is considered for analysis. The cross-section of the yarn is taken to be an elliptical shape with a 36.5-degree braiding angle. Afterward, the microscale and mesoscale finite element (FE) model is prepared, as presented in Figure 2. In the micro- and mesoscale modeling, it is assumed that the epoxy matrix is fully bonded with carbon fiber, and there are no voids between them. Additionally, the composite is pure, which means there are no impurities in it during the manufacturing process. Meshing is completed with C3D20R hexahedral elements, and meshing quality is also checked for better prediction [17]. The elements corresponding to the micro- and mesomeshed model are 99,431, and 384,741, respectively, with an element size of 0.01 mm. Element orientation is maintained for fiber bundles to control heterogeneity. Finite element analysis (FEA) is applied in Abaqus python code at the microscale, with matrix and fiber properties to calculate yarn properties; those are verified by the rule of mixture and Halpin–Tsai empirical formula according to Equations (1)–(4)

(1) $$E_{\mathrm{ii}}=V_{f}E_{f}+V_{m}E_{m},\;(i=1)$$

(2) EjjEm=1+ξ*η*Vf1−η*Vf, η=EfEm−1 EfEm+ξ, ξ=2, (j=2, 3)

(3) GijGm=1+ξ*η*Vf1−η*Vf, η=GfGm−1 GfEm+ξ, ξ=1, (i, j=1,2; 1,3; 2,3.)

(4) $$\upsilon_{\mathrm{ij}}=V_{f}\upsilon_{f}+V_{m}\upsilon_{m},\;(i,\;j=1,\;2;\;1,\;3;\;2,\;3.)$$

where *E*_ij_, *E*_jj_, *G*_ij_, and $\upsilon_{ij}$ are longitudinal, transverse, shear modulus, and Poission ratio. *E*_m_/*G*_m_, $\upsilon_{m}$, and *V*_m_ are matrix modulus, poission ratio, and voulme fraction. *E*_f_, *G*_f_, $\upsilon_{f}$, and *V*_f_ are fibers’ transverse and shear modulus, Poisson ratio, and volume fraction. ξ is the reinforcing efficiency, mainly depending on fiber geometry and packing; in some cases, such as circular fiber arrangements, it is considered as 2 for transverse modulus and 1 for shear modulus, and η is the average number called the stress partitioning factor, which varies between 0 to 1 [18].

Then, the mesoscale unit cell is analyzed to calculate the final effective stiffness with calculated yarn properties (microscale) and matrix material in Abaqus python code. Generally, the mechanical properties of braided composites can be described by nine independent elastic constants in the material stiffness matrix [19,20]. Analyses are performed by six displacement and periodic boundary conditions to obtain the material properties of the micro- and mesoscale. Face, edge, and vertex coupling are applied for pure tension and shear to propose the repeating nature. This can be achieved by classifying the node sets on the cell’s faces, edges, and vertices. In this condition, the displacement field under a macroscopic strain can be expressed as

(5) $$u_{i}\left(x_{1}{\;x}_{2}{\;x}_{3}\right)=u_{i}^{0}+u_{i}^{s}\left(x_{1}{\;x}_{2}{\;x}_{3}\right)$$

(6) $$u_{i}^{0}=\varepsilon_{\mathrm{ij}}^{0}x_{j}$$

In the above equations, Equation (6) $u_{i}^{0}=\varepsilon_{\mathrm{ij}}^{0}x_{j}$ represents the linear displacement field in the periodic composites. The modification of the linear displacement field is covered by the right side of Equation (5) ($u_{i}^{s}\left(x_{1}{\;x}_{2}{\;x}_{3}\right))$, due to the heterogeneity of composites, which is the periodic part of the boundary’s linear displacement field. The displacement field of Equations (1)–(2) can be applied on boundaries in parallel opposite pairs and can be written as $u_{i}^{k+}=$ $\varepsilon_{\mathrm{ij}}^{0}x_{j}^{k+}+u_{i}^{s}$ and, $u_{i}^{k-}=$ $\varepsilon_{\mathrm{ij}}^{0}x_{j}^{k-}+u_{i}^{s}$.

where k+ and k− specify the opposite parallel objects’ kth pair. Thus, due to periodicity, $u_{i}^{s}\;$is the same at the two opposite boundaries.

(7) $$u_{i}^{k+}-u_{i}^{k-}=\varepsilon_{ij}^{0}x_{j}^{k+}-x_{j}^{k-}=\varepsilon_{\mathrm{ij}}^{0}x_{j}^{k}$$.

Equation (7) denotes displacement variations between two parallel limitations (edge surface, corners) and can be called displacement periodic boundary conditions. Due to the constant nature of $x_{j}^{+}-x_{j}^{-}$ for nodes’ pair with a defined macroscopic strain, this can be applied in finite element analysis as nodal displacement conditions.

After applying all conditions and completing the analysis, postprocessing is performed to obtain the elastic modulus and Poisson ratio through E_11_ = σ_11_/ε_11_, E_22_ = σ_22_/ε_22_, E_33_ = σ_33_/ε_33_, G_12_ = τ_12_/γ_12_, G_13_ = τ_13_/γ_13_, G_23_ = τ_23_/γ_23_, ν_ij_ = ε_jj_/ε_ii_ (i, j = 1,2,3). Where 1, 2, and 3 stand for x, y, and z directions, respectively, followed by Equations (8)–(10).

(8) ε=Sσ

(9) $$\left[\begin{array}{c} \varepsilon_{11}\\ \varepsilon_{22}\\ \varepsilon_{33}\\ \gamma_{23}\\ \gamma_{13}\\ \gamma_{12} \end{array}\right]=\left(\begin{array}{cccccc} \frac{1}{E_{11}} & \frac{-\upsilon_{21}}{E_{22}} & \frac{-\upsilon_{31}}{E_{33}} & 0 & 0 & 0\\ \frac{-\upsilon_{12}}{E_{11}} & \frac{1}{E_{22}} & \frac{-\upsilon_{32}}{E_{33}} & 0 & 0 & 0\\ \frac{-\upsilon_{13}}{E_{11}} & \frac{-\upsilon_{23}}{E_{22}} & \frac{1}{E_{33}} & 0 & 0 & 0\\ 0 & 0 & 0 & \frac{1}{G_{23}} & S_{45} & 0\\ 0 & 0 & 0 & 0 & \frac{1}{G_{13}} & 0\\ 0 & 0 & 0 & 0 & 0 & \frac{1}{G_{12}} \end{array}\right)\left[\begin{array}{c} \sigma_{11}\\ \sigma_{22}\\ \sigma_{33}\\ \tau_{23}\\ \tau_{13}\\ \tau_{12} \end{array}\right]$$

(10) $$C_{\mathrm{ij}}=S_{\mathrm{ij}}^{-1}$$

The calculated stiffness for the braided epoxy carbon fiber reinforced plate is shown in Table 1. The calculated stiffness is verified with the existing literature’s experimental results. The literature has a 17.91 GPa out-of-the-plane direction, and the currently calculated stiffness has 17.75 Gpa, which shows a 0.89 percent error; this indicates that the calculated stiffness is significantly accurate [21].

**Figure 3:**
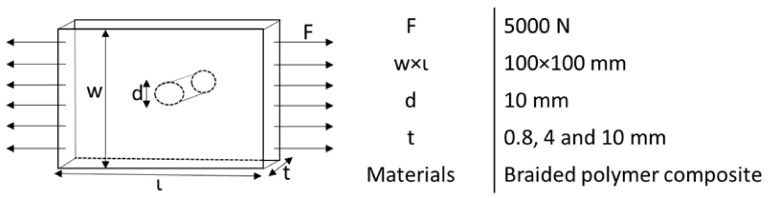
A basic model of the problem definition at the macroscale.

Finally, the macroscale homogenized plate is modeled. The macro plate properties are obtained by the combination of micro- and mesoscale analysis, so in the properties transfer process, it is considered that there is no information loss during coordinate transfer. The plate section is assigned by the calculated homogenized mechanical properties. The plate is subjected to a unidirectional tensile load of 5000 N with fixed support at the second end, as shown in Figure 3. The geometry and load are varied to collect various data for stress and SCF calculation.

### 2.2. Analysis of Macro Plate

**Figure 4:**
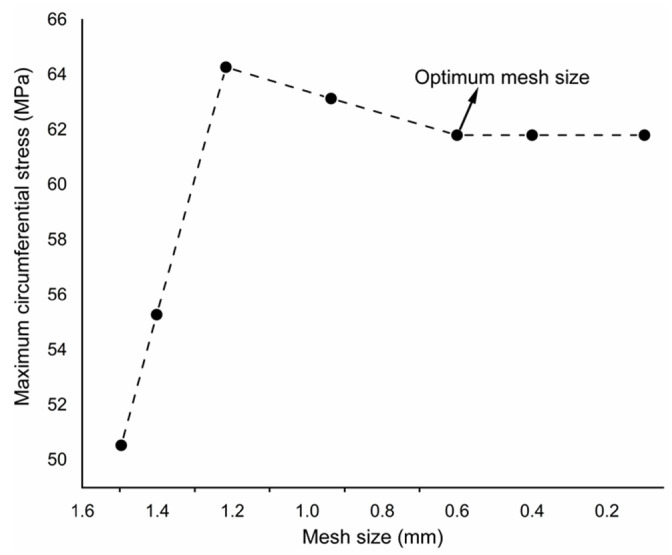
Mesh convergence for a braided plate with center hole.

**Figure 5:**
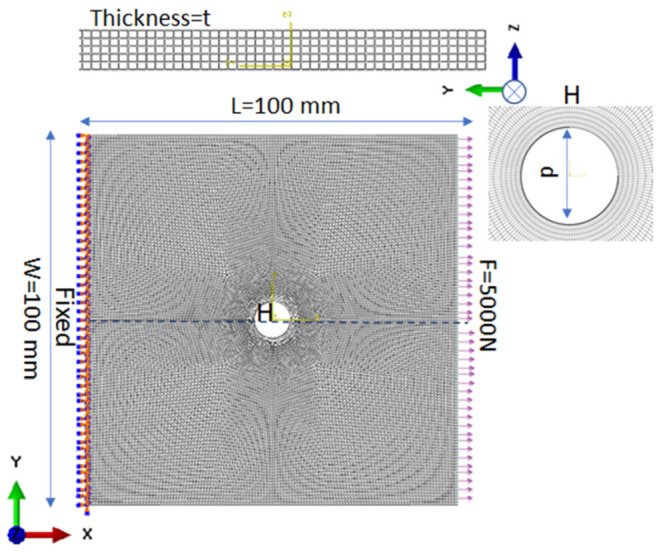
Finite element model of the macro plate.

The finite element method (FEM) is a strong computational approach for numerical modeling and optimization of structural geometry, especially when analytics is not possible, and experimentations are time and cost consuming [22]. The finite element analysis (FEA) is completed in the Abaqus commercial code to incorporate the modeling and analysis [23]. The thickness of the plate varies from thin, moderate thick to thick (0.8 mm, 4 mm, and 10 mm, respectively) according to the aspect ratio (0.008, 0.04, and 0.1, respectively) to establish the effectiveness of the theory. For each case, the length and width of the plate are equal to 100 mm and 100 mm, respectively, with a 10 mm center hole diameter. The plate is analyzed by first-order shear deformation theory (FSDT), higher-order shear deformation theory (HSDT; Reissner–Mindlin theory), and elasticity theory. Each thickness case is analyzed with all three theories. Mesh sensitivity analysis is performed to choose the optimum mesh size, and the maximum circumferential stress is analyzed for different mesh sizes (1.5, 1.4, 1.2, 1, and 0.6 mm), as shown in Figure 4. Then, after obtaining the mesh convergence at 0.6 mm size, mesh generation is completed with a universal element size of 0.6 mm, and the size of an element is minimized near the hole to capture the exact changes; the model is divided into four faces near the hole region with local sizes of 0.2, 0.3, and 0.4 mm far from the hole, respectively. It is refined near the hole to calculate accurate behavior. Finally, a mapped mesh is assigned to the plate and near the hole region to capture the exact geometry. The macro plate FE model is shown in Figure 5. The second-order reduced integration is chosen because second-order reduced integration predicts more accurate results than the corresponding fully integrated and first-order results. Additionally, it reduces running time, especially in a three-dimensional analysis. Firstly, stress distribution and concentration are calculated around a circular hole in a plate under uniaxial tension for the available literature conditions to verify the analysis process. Then, it is completed for the considered cases.

#### 2.2.1. Theoretical Formulations

The plate is defined as thin, thick, and moderate thick based on its dimensions. In a very thin plate, thickness is much less comparable to other dimensions, but in others, thickness has an influential role in dimensions.

(11) $$\frac{1}{10}\;\geq \;\frac{t}{w}\;\geq \;\frac{1}{2000}$$

*t* and *w* are thickness and other smallest dimensions of the plate, respectively. This paper adopts three theories for plate analysis: FSDT, HSDT, and elasticity theory. Equations (12)–(14) give the displacement field in the first-order shear deformation theory [24].

(12) $${u(x,y,z)=u}_{0}{(x,y)+z\psi }_{x}\left(x,y\right)$$

(13) $${v(x,y,z)=v}_{0}{(x,y)+z\psi }_{y}\left(x,y\right)$$

(14) w(x,y,z)=w(x,y)

where *u*, *v*, and *w*, are displacement components in the *x*, *y*, and *z* directions, respectively; $\psi_{x}$ and $\psi_{y}$ are rotations of the cross-section about the x and y axes, respectively; and u_0_ and v_0_ are displacement components in the plate’s mid-plane.

The displacements variations (*u*, *v*, and *w*) are given by the following Equations (15)–(18) as per the middle plane (*z* = 0) kinematics for HSDT (Reissner–Mindlin theoretical model) [25,26].

(15) $${u(x,y,z)=-z\theta }_{x}\left(x,y\right)$$

(16) $${v(x,y,z)=-z\theta }_{y}\left(x,y\right)$$

(17) $$w(x,y,z)=w(x,y)\;\mathrm{and}\;\theta_{x}=\frac{\partial w}{\partial x}+\phi_{x}\;\theta_{y}=\frac{\partial w}{\partial y}+\phi_{y}$$

where *x*, *y* is the in-plane and *z* are the transverse directions, and θ_x_ and θ_y_ are the angles of rotation for *xz* and *yz* plane, respectively, including extra rotation term about *x* and *y* axes because, after deformation, the normal plane is not really orthogonal to the middle plane.

(18) $$\underline{u}\left(x,y,z\right)=\begin{array}{c} u_{x}\left(x,y,z\right)\\ {\;u}_{y}\left(x,y,z\right)\\ u_{z}\left(x,y,z\right) \end{array}$$

In Equation (18), $\underline{u}\left(x,y,z\right)$ shows the displacement field in the elasticity theory, where $\underline{u}$ is the displacement vector, and $u_{x}$, $u_{y}$ and $u_{z}$ are projection of $\underline{u}$ on the x, y, and z axes. The elasticity theory is the 3D theory, which is close to an exact solution [27].

The theoretical calculation of stress distribution around a circular hole is performed based on Equations (19)–(21) for radial ($\sigma_{r})$, circumferential ($\sigma_{\theta })$, and radial–circumferential ($\sigma_{r\theta })$ directions in a plate under uniaxial tension.

(19) $$\sigma_{r}=\frac{F}{2}\left(1-\frac{a^{2}}{r^{2}}\right)-\frac{F}{2}\left(1-\frac{{4a}^{2}}{r^{2}}+\frac{{3a}^{4}}{r^{4}}\right)\cos2\theta$$

(20) $$\sigma_{\theta }=\frac{F}{2}\left(1+\frac{a^{2}}{r^{2}}\right)+\frac{F}{2}\left(1+\frac{{3a}^{4}}{r^{4}}\right)\cos2\theta$$

(21) $$\sigma_{r\theta }=-\frac{F}{2}\left(1+\frac{{2a}^{2}}{r^{2}}-\frac{{3a}^{4}}{r^{4}}\right)\sin2\theta$$

where *F* is the applied load, a is the radius, and r is the changing length from hole distance. The calculation of SCF is essential to calculate the maximum stress around the hole. According to Peterson [28], the SCF (K_T_) is specified as the ratio of extreme stress (σ_max_) in the hole zone under the actual loads to the nominal stress (σ_nom_) in the section, as shown in Equations (22)–(25) For the simple geometries, σ_max_ is assessed using numerical methods or analytical procedures. Experimental methods, such as photoelasticity or digital image correlation, can also be used to estimate it. In contrast, σ_nom_ can be computed with the help of the strength of materials formulae.

(22) $$K_{T\;}=\frac{\sigma_{\max}}{\sigma_{\mathrm{nom}}}$$

(23) $$\sigma_{\mathrm{nom}}=\frac{F}{t}*\left(w\;-\;d\right)$$

(24) $$\sigma_{\max}{=K}_{T}{*\sigma }_{\mathrm{nom}}$$

(25) $$K_{T}=3\;-\;3.13(\frac{d}{w})+3.66(\frac{d}{w}{)}^{2}\;-\;1.53(\frac{d}{w}{)}^{3}$$

The above-shown equation of SCF is for the isotropic plate. Equation (26) shows SCF for orthotropic cases where d/w is the hole diameter to plate width ratio.

(26) KT,o,p,u∞,1KT,o,p,u1=31 − dw2+1 − dw3+12dwM6KT,o,p,u ∞,1− 3*[(1 − (dwM)2]

$K_{T,o,p,u}^{\infty ,1}$ can be calculated by

(27) $$K_{T,o,p,u}^{\infty ,1}=1+\sqrt{2\left(\sqrt{\frac{E_{y}}{E_{x}}}{\;-\;\nu }_{\mathrm{yx}}+\frac{E_{y}}{{2G}_{\mathrm{yx}}}\right)}$$

M is the amplitude, which only depends on the geometry; for the case, the subsequent correlation is noticed for a finite-width plate.

(28) M2=1−831 − dw2+1 − dw3 − 12dw2

#### 2.2.2. Analysis Validation

**Figure 6:**
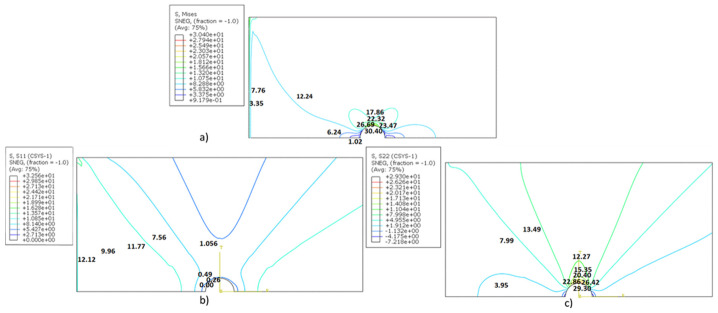
(**a**) Von Mises stress, (**b**) Radial stress, (**c**) Circumferential stress for the isotropic plate under uniform tension.

The analysis process is verified from the calculated results for isotropic/orthotropic plates because of the availability of theoretical results [29]. The isotropic plate’s Young’s modulus and Poisson ratio are 200 Gpa and 0.3. The theoretical maximum stress is 30.22 MPa, and the obtained maximum stress from the current analysis is 30.40 for an isotropic case, as shown in Figure 6. The error from the theoretical results is 0.6 percent. The radial and circumferential stress distributions are also compared with the theoretical formulation. The calculated variation behaves according to theoretical formulations (19)–(21). The distribution is shown in Figure 6b,c, which indicates there is no stress for the sigma radial, and the highest is at the hole radius point (θ = 90°) for circumferential stress, which, as per the theoretical variation, shows the accuracy of the calculation.

SCF is also calculated from theoretical calculations (Equations (22)–(28)) to validate the analysis process [29,30]. SCFs are 2.72 and 2.73 from theoretical calculations, and from current results they are 2.69 and 2.70, respectively; the error is 1.1 percent from the theoretical calculation. This shows that the current analysis process is significantly accurate. After verifying the analysis process, the results are calculated for the braided polymer plate.

## 3. Results and Discussion

**Figure 7:**
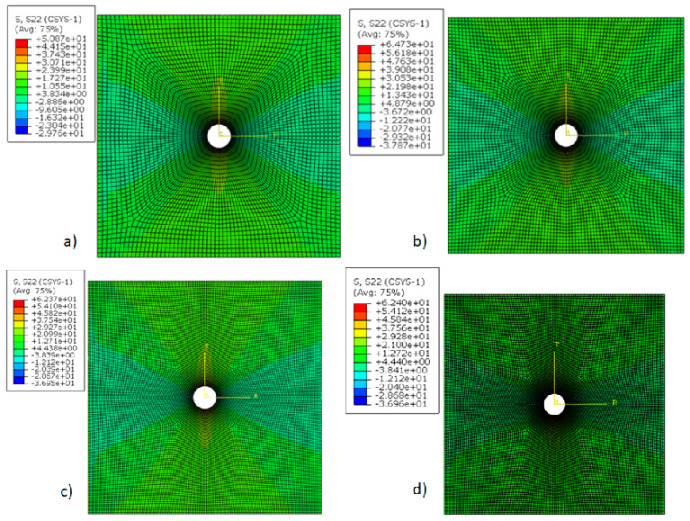
Mesh convergence analysis for mesh size (mm), (**a**) 1.5, (**b**) 1.2, (**c**) 0.6, (**d**) 0.2.

Studies on a plate with a hole have been conducted in previous research. The previous work deals with the stress and its concentration as a function of dimensions, loading, and the effects of boundary conditions for isotropic, laminated, and various composite materials. These have not been studied for a braided polymer plate. Additionally, the effects of analysis theory at different thicknesses were not studied. Therefore, the design estimation of a braided polymer plate with a center hole is presented. First, the multiscale modeling is conducted for a braided polymer plate to analyze its behavior. Then, the homogenized plate properties are obtained by carbon fiber and epoxy constituent for the numerical analysis; those are shown in Table 1. It is considered that there is exact information passed between the coordinates (micro to meso, then meso to macro) for the multiscale modeling. Multiscale modeling has another limitation: it is computationally expensive because it involves solving the subscale at each gauss point and load step. This is why it is taken care of by verifying the analysis process and results through the existing literature. Then, the macro analysis model is prepared, and its section is assigned with calculated mechanical properties for analysis. The optimum mesh size is selected after convergence of the mesh from different mesh sizes for an optimal result, as shown in Figure 4. The results of the mesh convergence analysis are shown in Figure 7. It can be seen that 0.6 mm is the optimal size for the macro plate because there is no difference between the hoop stress results (both are the same, about 62 MPa) of 0.6 mm and 0.2 mm, and 0.2 mm takes three times longer to perform the analysis. So, it does not make sense to choose a size denser than 0.6 mm. Moreover, the optimized mesh size (0.6 mm) is dense enough and covers the plates accurately.

Afterward, stress distributions are calculated; it is also stated to understand calculation requirements with the effectiveness of shear deformation theory at different thickness conditions. There are distinct calculation strategies for numerical analysis; it should be known which calculation process considers the accurate effect of stress for the available thickness aspect. So, FSDT, HSDT (Reissner–Mindlin theory), and elasticity theories are considered in the analysis for thin (aspect ratio 0.008), moderately thick (aspect ratio 0.04), and thick plates (aspect ratio 0.1). The analysis process is verified with the available literature data and then extended for cases of braided plates. Stress calculations are presented for analysis theories under variable thickness. It shows how the combined effect affects the stress distribution. Table 2 illustrates transverse stress and SCFs distribution for a braided polymer plate.

**Figure 8:**
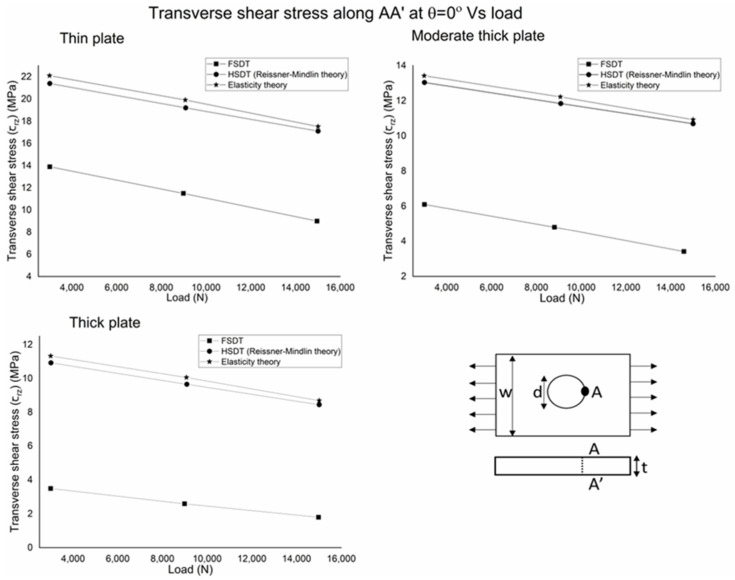
Transverse shear stress (ꞇ_rz_) variation with respect to uniaxial tension load.

**Figure 9:**
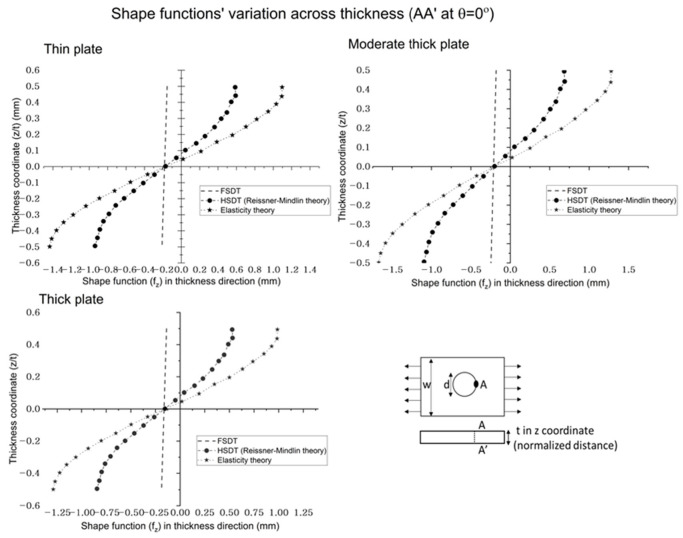
Shape function (f_z_) variation across the thickness of braided polymer plate.

Maximum transverse shear stress, calculated by FSDT, is 37, 56, and 70 percent less than HSDT and elasticity theory for thin, moderate thick, and thick plates, respectively. The results show that transverse stress depends on the analysis theories for variable thickness. It indicates that HSDT predicts similar results to the elasticity theory, as elasticity theory is the generalized 3D theory, so HSDT (Reissner–Mindlin) can predict accurate transverse shear stress variation, but FSDT is significantly different from the elasticity theory, so it cannot be considered for accurate transverse shear stress distribution. When the plate’s aspect ratio (L/t) is increased, the results’ difference of FSDT from HSDT and elasticity theory is increased. In addition, even transverse stress is significantly lower for thin plates, but HSDT is needed for accurate calculation because, although the plate is thin, the results’ difference is 35 percent, which is significantly high. It can also be seen in the transverse shear stress versus the load graph in Figure 8, which shows the variation of transverse stress with a change in load. The plot’s consideration point (across thickness A-A’) is at zero angle along the loading direction. The results indicate that when the load is increased, the transverse shear stress decreases; also, the decrease slope is the same for the analysis theories, but FSDT values are significantly lower than HSDT and the elasticity theory. Figure 9 shows the shape function with the normalized thickness coordinates at the center hole radius section; it indicates the sensitivity of calculations of analytical theories. It is foundthat the shape function of FSDT is a tilted linear line across the thickness, and HSDT curve behavior is the same as elasticity when calculating the behavior along with thickness for a braided polymer plate. The trend of the obtained results is verified by the existing literature. The transverse shear stress to load is checked to ensure that the trend of current results is the same as in the literature [31]. As the analysis findings for a polymer braided plate with center hole are conducted for the first time, only the analysis process and results trends are verified.

In the case of the effect on SCF, it is found that the maximum SCF is the same for similar theories and different thicknesses. Additionally, SCF varies with different theories, but the difference is minimal. For example, HSDT calculates as accurately as elasticity, but FSDT calculates about 3 percent less than the elasticity theory, as shown in Table 2. All three theories calculate the same maximum SCF for thin, moderately thick, and thick plates, but each theory has a different maximum value (2.53, 2.60, and 2.61, respectively), which shows the effectiveness of the HSDT theory.

**Figure 10:**
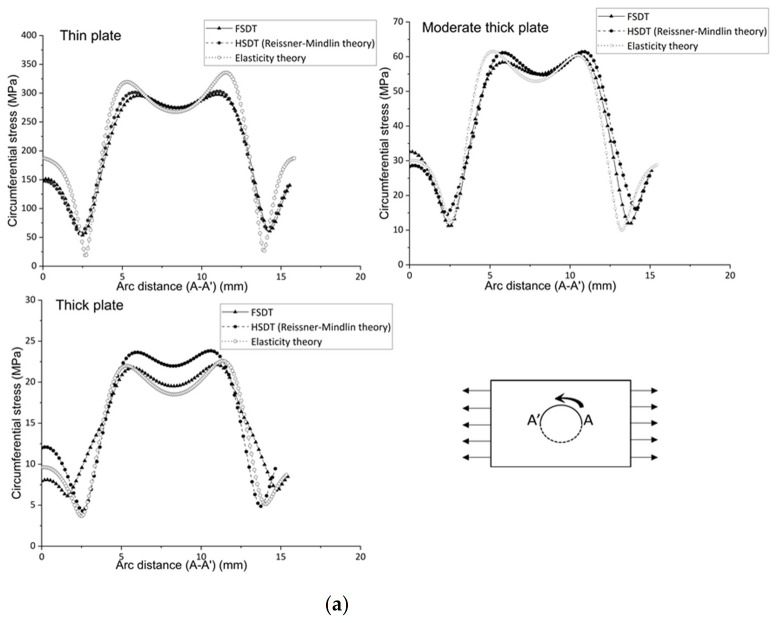

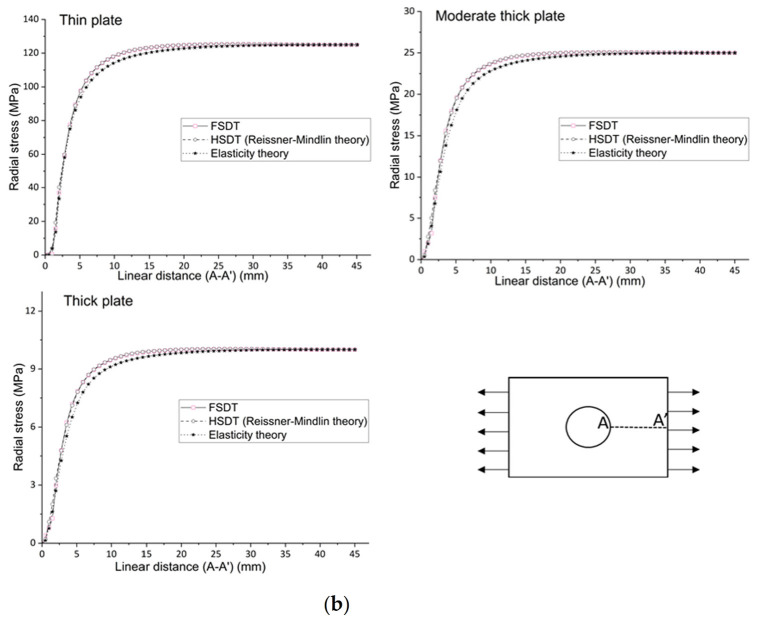
Stress variation around the hole radius for the braided polymer plate under uniaxial tension (**a**) Circumferential and (**b**) Radial.

Stress around the hole is also checked to analyze the combined effect. Below, Figure 10 a,b shows variations of circumferential and radial stress for different theories with varying thicknesses to check the in-plane stress variations.

It can be seen that hoop stress leads to higher stress slightly away from the middle of the hole radius in the braided plate due to its directional material behavior. The radial and circumferential stresses’ contour shows the stress variation around the plate hole. The hoop stress is oriented on the hole radius, but the concentration point is not exactly in the middle of the hole radius; the variation of the curve is like the top (at the left side point A’), down (middle point), and top (right side point A) around the hole radius, as shown in Figure 10. The radial stress varies from zero to maximum from the hole radius to the edge point. First, the values increase linearly and then curve and are finally constant until the edge. All theories have a similar trend for each thickness to calculate the radial stress. In addition, FSDT and HSDT are as accurate as the elasticity theory; the error difference is only 1.6 percent for each thickness aspect. For circumferential stress, FSDT and HSDT obtain the same results as the elasticity theory for thin and moderately thick plates, but for thick plates, FSDT and HSDT obtain 1.5 and 0.4 percent errors, respectively, compared to the elasticity theory, which is minimal. So, to calculate the radial and circumferential stress, it is good practice to choose FSDT because of less computation and modeling time. Thus, if there is consideration other than transverse shear stress (such as circumferential, radial stresses, and other plane stresses in primary consideration), then FSDT can be considered to save time and cost, but consideration of the transverse shear stress HSDT is needed.

**Figure 11:**
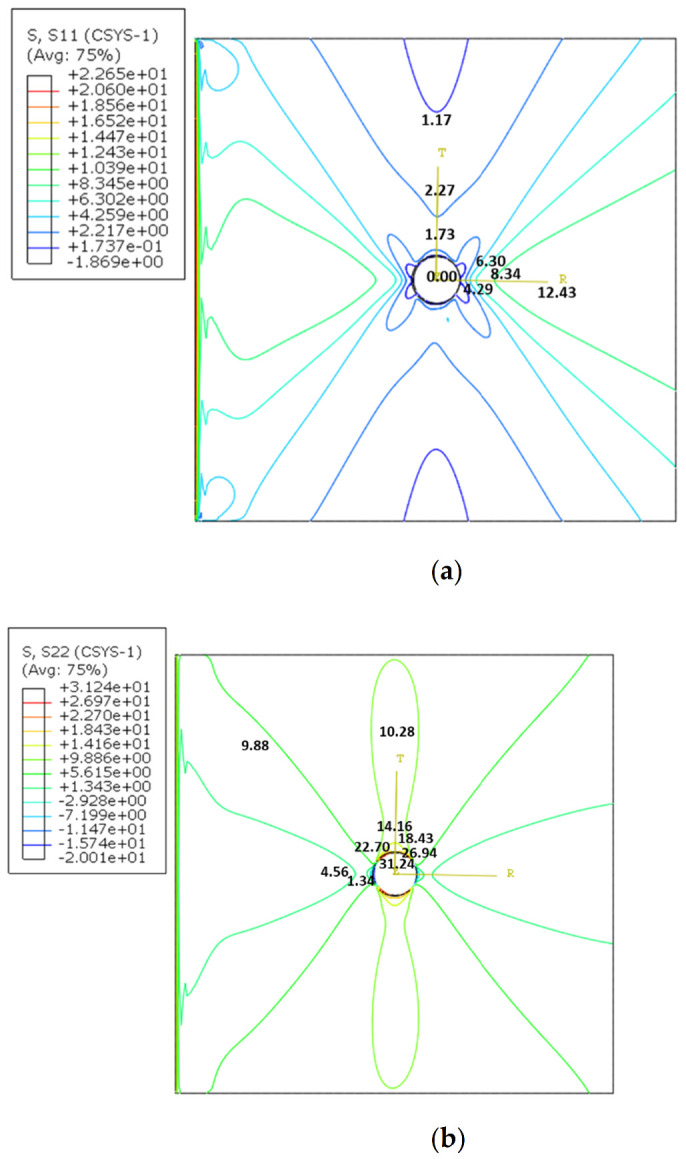
Stress contour distribution in the braided plate under uniform tension (**a**) Radial and (**b**) circumferential.

Radial and circumferential (hoop) stresses’ contours are presented in Figure 11 to show stress variation for a braided plate through the elasticity theory to represent the exactness of results. The contour values show that stress is zero and maximum at ninety degrees of load direction for radial and circumferential stresses, respectively, as per the theoretical formation of stress distribution in a rectangular/square plate with a hole under tensile load. In addition, radial stresses are increasing away from the hole, and circumferential stresses are highest around the hole, verifying the analysis process. Therefore, the contour value and representation of stress can be taken as a reference for predesigning braided polymer plates with a hole. So, while designing with open-hole situations, such as fasteners or bolts for a 3D braided polymer plate, the stress situations presented could be considered for prestressing the area.

Additionally, computation time should be known for material conditions, approximately 2–3 times higher in the braided plate case than isotropic/orthotropic calculations, with 64 GB ram and 3.2 GHz processor. Likewise, computation time should be known for all theories; for FSDT, HSDT, and elasticity theory, this is 10 min, 25 min, and 3–4 h, with 64 GB ram and 3.2 Ghz processor. So, one can choose the analysis theory wisely according to the interest of the calculation and model. As braiding yarns are formed cyclically, and the repeating part is chosen according to the manufacturing process, the uniformity of location is maintained in the analysis. Additionally, the braiding angle is not varied in the current analyses because the lower braiding angle has higher stiffness than the higher braiding angle. So, the consideration of variation in the braiding angle, boundary, and load conditions will be performed in the future.

**Figure 12:**
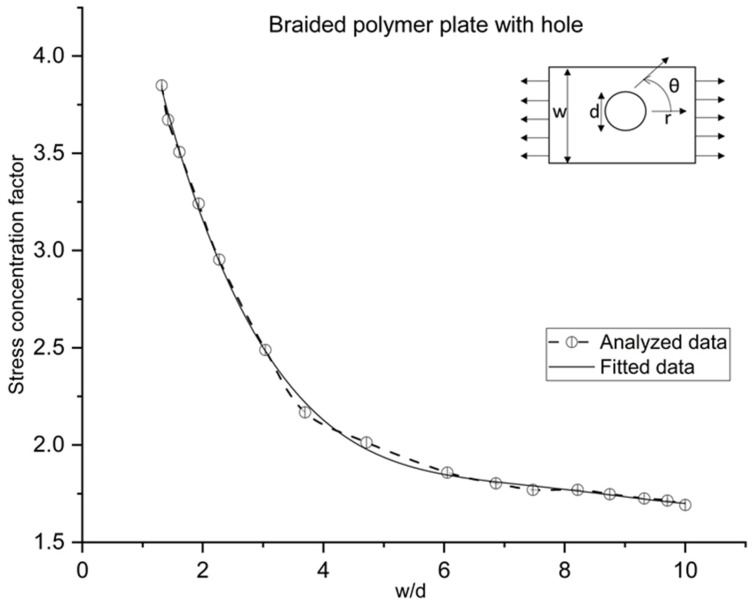
Effect of w/d on SCF in braided polymer plate under uniform tension for circumferential stress.

Moreover, the SCF variations with width to hole diameter ratio are also examined for a braided plate, as shown in Figure 12. First, the SCF is decreased continuously in a curve form, from 3.84 to 1.85 for 1.32 to 6.05 width to hole diameter (w/d); then, the value of SCF is approximately constant (1.77) until 8.7 w/d, then linearly decreasing a little from 1.77 to 1.69 for 8.7 to 10 w/d ratio. According to SCF behavior for the braided plate for w/d, the formulation is performed for future design correspondence, shown in Equation (29). The current trend is validated by the literature, which illustrates the SCFs variation for an orthotropic plate with a hole [30].

(29) $$\begin{array}{c} Braided\_Polymer_{\mathrm{SCF}}\left(\frac{w}{d}\right)\\ =5.82232-1.93314*\left(w/d\right)+0.35496*{\left(\frac{w}{d}\right)}^{2}-0.02916*{\left(w/d\right)}^{3}+8.79125E-4\\ {}*{\left(\frac{w}{d}\right)}^{4}+8.39971E-7\;*{\left(w/d\right)}^{5} \end{array}$$

Thus, the current design study will provide the pre-estimation of designing the braided polymer plate with a hole due to bolt, fastener, or other conditions.

## 4. Conclusions

Small geometric changes in plates result in sharp differences in mechanical characteristics; a hole in a plate is a topic of research to analyze its effect because this kind of structure is commonly used in many places, such as aero, automobiles, marine, etc. Many researchers have reported the effects of factors such as length, diameter, isotropic, and laminates, etc., but this has not yet been conducted for braided polymer plates. Additionally, the combined effect of calculation theories for different thickness aspect ratios have not been studied. The selection of stress calculation theories is an important criterion for particular thickness conditions. This paper investigates the mechanical behavior of a braided polymer composite plate to know the design aspects by considering shear deformation theories for variable thicknesses. In addition, current research predicts the SCF formulation with respect to the w/d ratio of a braided polymer plate. First, multiscale modeling is conducted to calculate mechanical stiffness and then applied to analyze the macro part. The analysis model is set up, and the process is performed and verified at every stage. Shear deformation theory’s effects are examined on circumferential, radial, and transverse shear stress with SCF under the variable thickness of the braided plate. Some important findings are:

- Hoop stress variation is like a Z curve shape around the central circular hole.
- The SCF is varied like a concave curve, decreasing while increasing plate width to hole diameter ratio (w/d).
- FSDT calculates 37, 56, and 70 percent less maximum transverse shear stress than HSDT and elasticity theory for thin, moderately thick, and thick plates for a braided polymer plate. Thus, FSDT cannot calculate accurate transverse stress, but HSDT (Reissner–Mindlin) is as accurate as the elasticity theory.
- The change in theory does not affect the circumferential stress, radial stress, and SCF. However, HSDT has higher accuracy than FSDT because FSDT has an approximate 3 percent error from elasticity theory to calculate SCF, but HSDT has an approximate 0.5 percent error. Additionally, the error difference is not significant and can be under a considerable limit. Therefore, after knowing the time and modeling cost of HSDT and elasticity theory, which is 3–4 and 15–20 times higher than FSDT, if transverse shear stress is not under consideration, FSDT is suggested; otherwise, HSDT is required.
- The formulation of SCF of a braided polymer plate showing less curve variation means less stress concentration than the equivalent isotropic/orthotropic plate with a center hole.

Overall, this research covers the design analysis of a braided polymer plate with a hole for industrial and research applications. In the future, it needs to perform versatile models and analyses with different boundary, braiding, load, and analysis theories conditions to cover multipurpose behavior in one place for braided polymer plates with center and arbitrary holes for establishing a versatile design environment. This research will be helpful for researchers working in the same domain.

## Figures and Tables

**Table 1:** Material properties used in the unit cell model in microscale.

Constituent. ↓	Properties→	Young Modulus (GPa)			Shear Modulus (GPa)			Poisson Ratio		
		E_x_	E_y_	E_z_	G_xy_	G_xz_	G_yz_	ν_xy_	ν_xz_	ν_yz_
Fiber	Carbon T300	230	40	40	24	24	14.3	0.26	0.26	0.399
matrix	Epoxy resin	3.5						0.35		
Yarn (fiber volume fraction (0.52))	Microscale homogenizations (microscale unit cell)	121.28	10.16	10.16	7.93	7.93	4.36	0.303	0.303	0.375
Braided polymer plate with a center hole	Mesoscale homogenizations (mesoscale unit cell)	$\left[\begin{array}{c} 15.78\;\\ 7.35\\ 14.25\\ 0\\ 0\\ 0 \end{array}\;\begin{array}{c} 7.38\;\\ \;15.73\\ 14.27\\ 0\\ 0\\ 0 \end{array}\begin{array}{c} 8.55\\ 8.53\\ \;28.29\\ 0\\ 0\\ 0 \end{array}\begin{array}{c} 0\\ 0\\ 0\\ 18.25\\ 0\\ 0 \end{array}\begin{array}{c} 0\\ 0\\ 0\\ 0\\ 18.21\\ 0 \end{array}\begin{array}{c} 0\\ 0\\ 0\\ 0\\ 0\\ 11.55 \end{array}\right]$								

**Table 2:** Transverse shear stress and SCF for a braided polymer material.

Analysis Conditions (Plate Thickness_Analysis Theory)		Maximum Transverse Shear Stress (MPa)		Stress Concentration Factor (Maximum)
		(ꞇ_rz_) (0,w/2,0)	(ꞇ_θ__z_) (l/2,0,0)	
Thin plate (aspect ratio 0.008)	FSDT	13.61	3.78	2.53
	HSDT (Reissner–Mindlin theory)	21.50	5.90	2.60
	Elasticity theory	21.74	6.06	2.61
Moderate thick plate (aspect ratio 0.04)	FSDT	5.63	2.06	2.53
	HSDT (Reissner–Mindlin theory)	12.78	4.50	2.60
	Elasticity theory	12.82	4.75	2.61
Thick plate (aspect ratio 0.1)	FSDT	3.14	0.95	2.53
	HSDT (Reissner–Mindlin theory)	10.56	3.08	2.60
	Elasticity theory	9.30	3.1	2.61

## Data Availability

The data presented in this study are available on request from the corresponding author.

## Nomenclature

E	Longitudinal modulus
G	Shear modulus
υ	Poisson ratio
V	Volume fraction
σ, and ꞇ	Longitudinal and shear stress
ε, and γ	Longitudinal and shear strain
S	Compliance matrix
C	Stiffness matrix
ξ	Reinforcing efficiency
η	Stress partitioning factor
$u_{i}^{0}$	Linear displacement field in the periodic composites
t	Thickness of plate
w	Width of plate
F	Applied force
$\sigma_{r}$	Radial
$\sigma_{\theta }$	Circumferential
$\sigma_{r\theta }$	Radial–circumferential
K	Young’s modulus
d	Shear modulus
ꞇ_rz_, and **σ**_θz_	Transverse shear stress in cylindrical coordiantes
$\psi_{x}$, and $\psi_{y}$	Rotations of the cross-section about the x and y axes
θ_x_, and θ_y_	Angles of the rotation for xz and yz plane
